# DATASAPROX: standardised dataset from protocolled sampling of saproxylic beetles in continental France and Corsica

**DOI:** 10.3897/BDJ.14.e187021

**Published:** 2026-03-09

**Authors:** Christopher Bosc, Julien Touroult, Arnaud Horellou, Bruno Mériguet, Antoine Brin, Lionel Valladares, Hervé Brustel, Thomas Barnouin, Fabien Soldati, Olivier Delzons, Florian Buralli, Yoan Braud, Benjamin Calmont, Franck Herbrecht, Sébastien Etienne, Alain Roques, Nicolas Gouix, Nicolas Moulin, Benoît Dodelin, Guillaume Delporte, Jacques Pages, Romain Chambord, Laurent Chabrol, Jean-Hervé Yvinec, Christophe Bouget

**Affiliations:** 1 INRAE EFNO, Nogent-sur-Vernisson, France INRAE EFNO Nogent-sur-Vernisson France; 2 Patrinat, Paris, France Patrinat Paris France; 3 OPIE, Guyancourt Cedex, France OPIE Guyancourt Cedex France; 4 INP PURPAN, Toulouse, France INP PURPAN Toulouse France; 5 ONF, Quillan, France ONF Quillan France; 6 CEN PACA, Aix-en-Provence, France CEN PACA Aix-en-Provence France; 7 SHNAO, Aubière, France SHNAO Aubière France; 8 GRETIA, Rennes, France GRETIA Rennes France; 9 INRAE URZF, Orleans Cedex 2, France INRAE URZF Orleans Cedex 2 France; 10 CEN Occitanie, Montpellier, France CEN Occitanie Montpellier France; 11 Freelance entomologist, Montérolier, France Freelance entomologist Montérolier France; 12 Freelance entomologist, Lyon, France Freelance entomologist Lyon France; 13 GON, Lille, France GON Lille France; 14 BioDev'mlhl, Rosis, France BioDev'mlhl Rosis France; 15 SEL, Ussel, France SEL Ussel France; 16 ADEP, Bienville, France ADEP Bienville France; 17 Freelance entomologist, Bienville, France Freelance entomologist Bienville France

**Keywords:** flight interception traps, occurrence, saproxylic beetles

## Abstract

**Background:**

As part of the DATASAPROX project, we created an occurrence dataset of the saproxylic beetles from continental France and Corsica focused on protocolled data from flight interception traps. For this, we solicited the main national data producers and collected their raw data as well as the associated trapping metadata (e.g. type of trap). Once retrieved, all data went through selection and reformatting steps, including the removal of duplicates between datasets and the standardisation of taxonomic information according to the TAXREF v.18 format. We considered data from specific aerial interception traps: crossed window (©Polytrap, ©Pimul, ©Crosstrap, ©Portrap), multi-funnel (Lindgren) and window (single glass) traps.

**New information:**

We aggregated a dataset constituting 675,525 records from 21 data producers and providers, including 485 identified projects. This dataset includes a total of 71 families, 153 subfamilies, 738 genera and 2039 species of saproxylic beetles. Data points encompass all 96 counties from continental France and Corsica for a total of 8969 geographic points and 66,202 samples. This new standardised dataset on saproxylic beetles offers new opportunities for ecological studies and conservation applications.

## Introduction

Saproxylic beetles can be defined as Coleoptera which depend on dead or decaying wood for at least a part of their life cycle and, more specifically, on lignified tissue from trees and shrubs (Alexander 2008, Bouget et al. 2019). The group includes diverse feeding guilds, such as xylomycetophagous, myrmecophilous, xylophagous, saproxylophagous, predatory and parasitoid taxa, as well as taxa associated with sap, tree cavities and cones. They all have specific ecological requirements which are more likely to be met in forests, including ancient ageing trees offering a variety of microhabitats (e.g. dead or decaying aerial branches, cavities), which makes them highly sensitive to spatio-temporal changes of their habitat (Grove 2002, Alexander 2008).

They constitute intricate and highly specialised food webs in those ecosystems (Wende et al. 2017). They are essential contributors to deadwood decomposition and the recycling of organic matter (Chen and Forschler 2016, Kahl et al. 2017) and also represent a substantial resource to many consumers, such as insectivorous birds (Otvos 1979, Hebda et al. 2019) or parasitoid wasps (Hedgren 2007, Gibb et al. 2008, Vogel et al. 2021). In addition, many species with saproxylic larvae are floricolous at the adult stage and contribute to forest plant pollination. As such, their presence in woody areas sustains diverse ecosystems and more globally healthy forests.

In France, there are 2663 species of obligatory and facultative saproxylic beetles encompassing 74 families, which represents about a quarter of forest species (Bouget et al. 2019). However, saproxylic beetles have globally been subjected to various human-related threats, including deforestation, habitat loss and fragmentation (Bouget and Parmain 2016), overexploitation for wood harvest (Lassauce et al. 2012, Larrieu et al. 2012, Bouget et al. 2014, de Groot et al. 2016, Bouget et al. 2021), importation of exotic trees (Ulyshen et al. 2018), invasive species (Soldati et al. 2024) and climate change (Vitali et al. 2023). Hence, in Europe, 21.7% of species are considered threatened (Calix et al. 2018). Appropriate conservation measures are thus urgently needed, but their success depends notably on the acquisition of informative data on saproxylic beetle populations.

For that purpose, in France, the SAPROX survey programme has been carried out with the aim of gathering data on the biology and distribution of species and making it available to entomologists and to forest and natural reserve managers. The acquisition of these data on saproxylic beetles is instrumental for identifying hotspots in terms of biodiversity and conservation, tracking population changes in the face of environmental changes, but also for estimating the efficacy of policies for sustainable forest management practices, such as those favouring the retention and maintenance of dead wood and old trees (Grove 2002).

### Purpose

The SAPROX programme elicited agencies in charge of forest biodiversity and management, as well as research departments and independent entomologists, to share saproxylic beetle data collected using a diversity of methods, ranging from opportunistic observations to standardised sampling using flight interception traps. Flight interception trap sampling is recognised as an effective passive method to collect saproxylic beetle communities (Bouget et al. 2008, Bouget et al. 2009, Nageleisen and Bouget 2009a), with the Polytrap model being the most popular amongst entomologists for its selectivity and reliability (Brustel 2012). A large amount of these data has been gathered and integrated into the SINP, the information system from the National Inventory of Natural Patrimony (INPN). The data are curated and standardised using the French Taxonomic Referential (TAXREF; MNHN (2025)) that registers the valid scientific name of all species inventoried in metropolitan France. SINP is the French equivalent of GBIF and the two systems regularly exchange data.

On the SINP platform, for some datasets, the sampling method has not been detailed or sometimes not been provided by the data producer, which complicates their exploitation for ecological analyses, notably the estimation of biodiversity patterns and the study of biogeographical gradients, which require systematic and standardised community-wide samplings.

This is why, as a follow-up to the SAPROX programme, the DATASAPROX project has been carried out with the aim of creating a dataset, focused specifically on the standardised – protocolled – sampling part of saproxylic beetle data, i.e. on data from flight interception traps, the most widely used sampling technique. This necessitated fetching the raw data directly from the producers in order to ensure the acquisition of data from flight interception traps associated with explicit metadata on the sampling protocol.

The project resulted in the constitution of the present standardised dataset of the saproxylic beetles of continental France and Corsica, which we hope will offer new opportunities for ecological studies, as well as further applications in terms of conservation.

## Project description

### Title

DATASAPROX: standardised dataset from protocolled sampling of saproxylic beetles in continental France and Corsica

### Personnel

A total of 21 data producers involved in SAPROX and/or SINP provided their raw data. Those include national and regional agencies as well as independent entomologists. In the present dataset, they are mentioned using codes (in the “ownerInstitutionCode” field). Table 1 presents the corresponding names.

## Sampling methods

### Sampling description

In order to obtain a standardised dataset in terms of methods, we only selected specific flight interception trap types (details in Table 2) known to effectively target the saproxylic beetle community, i.e. cross-vane window flight traps (models: polytrap, pimul) (Brustel 2012), cross-vane panel flight traps (models: crosstrap, portrap) (Jurc et al. 2016), multi-funnel flight traps (model: lindgren) (Rassati et al. 2019) and single-vane window flight traps. These traps are usually transparent (window traps) or black (multifunnel and panel traps), sometimes a mix of both, but can also present alternative colours. They can also be supplemented with chemical baits (alcohol, terpenes or pheromones) and be associated with specific accessories, such as mesh protections. We did not include data from eclector, emergence, malaise, coloured pan or pitfall traps, as those are usually non-standardised methods with relatively poor selectivity towards saproxylic beetle taxa (Nageleisen and Bouget 2009a).

### Quality control

This dataset reunites different raw datasets from different sources (producers) across metropolitan France and Corsica, which involved several steps to harmonise field names and metadata. The dataset structure was created with the help of R software.

The type of occurrence data was usually assigned, based on information from the producer. Qualitative data has furthermore been automatically identified in the dataset on a per-project basis. That is, for each project (identified in the datasetName field), if all sampling events included only abundances = 1, all the records for the project were considered presences (organismQuantityType = presence).

Spatial coordinates were all transformed to the WGS84 projection system (EPSG: 4326). Information on administration (municipality, county) and altitude were then obtained for all the data with the BD TOPO and BD_ALTI map layers, retrieved from the French Institute for Geographic Information (IGN) web portal.

We only considered data associated with at least a valid and precise date (year-month-day), corresponding to the end of the sampling period. The date of the start of sampling was not always provided by the producers and so the sampling duration was not always directly deductible. In that case, we estimated the sampling duration by calculating the difference between the end date of the current sampling session and the previous one, knowing that there are usually several uninterrupted sampling sessions per year. If not applicable (e.g. only one sampling session), duration was given as the average at the project (datasetName) level. Sampling period is always between March and October of the same year.

Based on the taxon names provided by the producers, we retrieved taxonomical information according to the French TAXREF taxonomical convention (v.18) (“TAXREF” 2025), that is family, subfamily and scientific name (generic and species names, author and year), as well as the unique reference name identifier of the taxon (CD_NOM). We considered only saproxylic beetles identified at the species, genus, subfamily and family level (list of saproxylic beetle species derived from Bouget et al. (2019)). During the process, new exotic taxa were added to the list. These do not yet possess a name identifier (CD_NOM) within the TAXREF convention.

We identified and removed duplicated data entries between producer datasets. For each pair of producer datasets (identified in the ownerInstitutionCode field), we identified sampling events with comparable geographic coordinates and dates and including records with similar taxa names and abundances. Once identified, the duplicated sampling events were removed from one of the producer datasets, which we selected, based on the most recent update or based on metadata completeness (we always kept the most recent and/or complete data within each indentified sampling event).

For some producer datasets, we redirected metadata included into general remarks fields into new, more explicit fields, such as Location, ID_plot, ID_subplot or trap_name.

As our aim was to gather only data on species communities, projects focused on only one species were not included in our dataset.

## Geographic coverage

### Description

**Figure 1.**
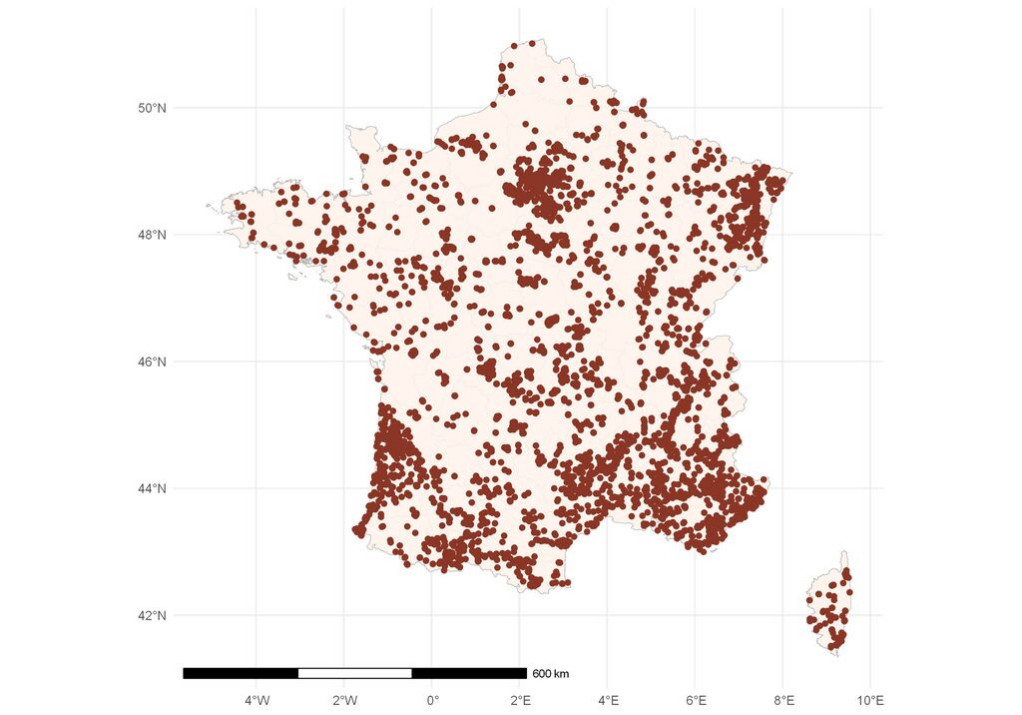
Map of the sampling points included in the dataset.

The present dataset includes only saproxylic beetles sampled in continental France and Corsica and encompasses all the 96 counties (Fig. 1). This represents a total of 8969 geographic points and 66,202 samples.

### Coordinates

41.49689 and 51.01095 Latitude; -4.42961 and 9.52743 Longitude.

## Taxonomic coverage

### Description

This dataset includes a total of 71 families (see Table 3), 153 subfamilies, 738 genera and 2039 species of saproxylic beetles. This dataset represents about 77% of the species recorded on the French list of saproxylic Coleoptera (Bouget et al. 2019), updated with new exotic taxa recently added to the list. We can note, however, in Table 3, discrepancies of representativity between families. Notably, while the Staphylinidae is the family with the highest number of known saproxylic beetle species in France, our dataset includes only 61% of those, while the Curculionidae Scolytinae and the Cerambycidae, two other speciose groups, are well-represented with 90% of the known French species. The Curculionidae records concern, to a great majority, the Scolytinae subfamily (93%). This is by far the most represented group in the dataset (20% of records).

### Taxa included

**Table:** 

Rank	Scientific Name	
family	Thymalidae	
family	Lycidae	
family	Elmidae	
family	Lymexylidae	
family	Zopheridae	
family	Cerophytidae	
family	Erotylidae	
family	Phloiophilidae	
family	Trogossitidae	
family	Clambidae	
family	Dryopidae	
family	Trogidae	
family	Histeridae	
family	Cryptophagidae	
family	Rhysodidae	
family	Melyridae	
family	Sphindidae	
family	Mycetophagidae	
family	Biphyllidae	
family	Carabidae	
family	Lophocateridae	
family	Buprestidae	
family	Phloeostichidae	
family	Curculionidae	
family	Scraptiidae	
family	Cerylonidae	
family	Ptiliidae	
family	Alexiidae	
family	Endomychidae	
family	Bostrichidae	
family	Latridiidae	
family	Staphylinidae	
family	Elateridae	
family	Rhadalidae	
family	Salpingidae	
family	Peltidae	
family	Tetratomidae	
family	Dryophthoridae	
family	Throscidae	
family	Mordellidae	
family	Ptinidae	
family	Cucujidae	
family	Nosodendridae	
family	Hydrophilidae	
family	Eucinetidae	
family	Cantharidae	
family	Melandryidae	
family	Bothrideridae	
family	Scarabaeidae	
family	Oedemeridae	
family	Corylophidae	
family	Silvanidae	
family	Pyrochroidae	
family	Aderidae	
family	Ciidae	
family	Dermestidae	
family	Monotomidae	
family	Leiodidae	
family	Cleridae	
family	Anthribidae	
family	Scirtidae	
family	Cerambycidae	
family	Sphaeritidae	
family	Prostomidae	
family	Brentidae	
family	Tenebrionidae	
family	Lucanidae	
family	Nitidulidae	
family	Pythidae	
family	Laemophloeidae	
family	Eucnemidae	

## Temporal coverage

### Notes

**Figure 2.**
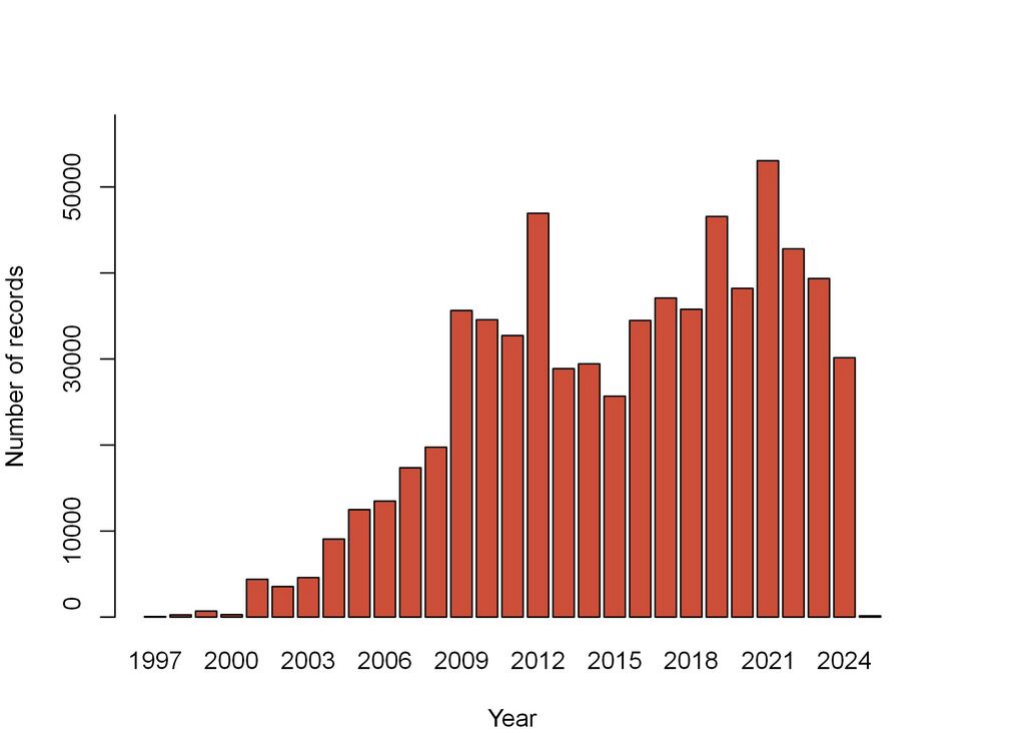
Number of records per year in the dataset.

**Figure 3.**
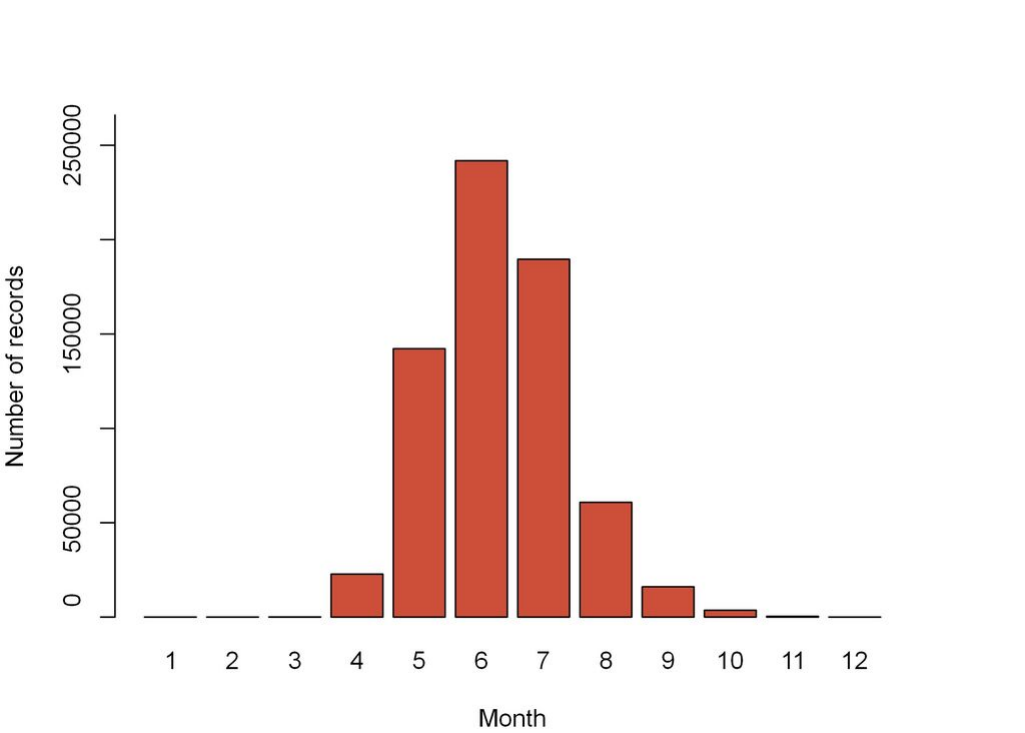
Number of records per month in the dataset.

The charts below (Figs 2, 3) display the distribution of data across the years and months of the year. We can see in Fig. 2 that the amount of data collected with flight interception traps rises in the 2000s, through the marketing of easy-to-use trap models. The collection months (Fig. 3) correspond to the activity period of most saproxylic beetle species (Nageleisen and Bouget 2009b).

## Usage licence

### Usage licence

Other

### IP rights notes

This work is licensed under a Creative Commons Attribution (CC-BY 4.0) Licence.

## Data resources

### Data package title

DATASAPROX: standardised dataset of saproxylic beetles from flight interception traps in continental France and Corsica

### Resource link

https://doi.org/10.15468/7xhf88

### Alternative identifiers

https://ipt.gbif.fr/resource?r=datasaprox

### Number of data sets

1

### Data set 1.

#### Data set name

DATASAPROX: standardised dataset of saproxylic beetles from flight interception traps in continental France and Corsica

#### Data format

Darwin Core

#### Download URL

https://ipt.gbif.fr/archive.do?r=datasaprox&v=1.6

#### Description

As part of the DATASAPROX project, we created an occurrence dataset of the saproxylic beetles from continental France and Corsica focused on protocolled data from flight interception traps (Bosc et al. 2025). For this, we solicited the main national data producers and collected their raw data as well as the associated trapping metadata (e.g. type of trap). Once retrieved, all data went through selection and reformatting steps, such as removing duplicates between datasets and standardising taxonomic information according to the TAXREF v.18 format. We considered data from aerial interception traps of the 'crossed window' type (©Polytrap, ©Pimul, ©Crosstrap, ©Portrap), multi-funnel (Lindgren) and window (single glass) traps.

**Data set 1.** 

Column label	Column description
ownerInstitutionCode	Code of the person or organism which produced and provided the data.
catalogNumber	Unique record identifier, attributed to a data entry in the original producer dataset, before data selection, curation and integration in the final dataset.
datasetName	name of the project or study associated with the data, provided by the data producer.
occurrenceID	Unique identifier of the occurrence record. Can be provided by the data producer, otherwise correspond to the catalogNumber.
eventID	Unique sample identifier.
family	Family name.
subfamily	Subfamily name.
genericName	Generic name.
taxonID	Unique taxon name identifier from the French TAXREF v.18 taxonomical convention (CD_NOM).
scientificName	Valid taxon name (family, subfamily, genus or species) according to the French TAXREF v.18 taxonomical convention.
taxonRank	Taxonomical rank of the taxon. We only included saproxylic Coleoptera identified at the species, genus, subfamily or family level.
organismQuantity	Number of individuals (abundance; quantitative data) or presence (qualitative data). Quantitative and qualitative data are differentiated in the complementary field “organismQuantityType”.
organismQuantityType	Type of occurrence data. Two possible values: individuals (quantitative data); presence (qualitative data).
eventDate	Date (year-month-day) of the end of sampling (trapping).
month	Month at the end of sampling (trapping).
year	Year at the end of sampling (trapping).
sampleSizeValue	Estimated sampling (trapping) duration.
sampleSizeUnit	Unit of the sampling (trapping) duration provided in the field “sampleSizeValue” (day).
decimalLongitude	Longitude of the trap in WGS84.
decimalLatitude	Latitude of the trap in WGS84.
georeferenceRemarks	Indicates if the given GPS coordinates correspond to a point or to a centroid of a larger area. Possible values: *point*; *centroid.*
coordinateUncertaintyInMetres	Level of geographical precision in metres.
municipality	Name of the municipality in which the sampling took place.
county	Name of the county (French Department) in which the sampling took place.
maximumElevationInMetres	Maximum altitude in metres, extracted from map layers, based on the GPS coordinates.
verbatimElevation	Altitude as given in the producer dataset.
minimumElevationInMetres	Minimum altitude in metres, extracted from map layers, based on the GPS coordinates.
dynamicProperties	Information on the sampling design and the flight interception trap. Several concatenated fields in JSON format: ID_plot: Identifier of the sampled plot; ID_subplot: Identifier of the sampled quadrat (included within a plot); trap_num: Number identifying the trap; trap_name: Code or name identifying the trap; trap_stratum: Vertical position of the trap in the vegetation; ID_condition: Treatments of the sampling design; trap_model: Model of the trap; trap_type: Type of the trap; trap_colour: Main colour of the trap; trap_bait: Type of chemical bait used with the trap; trap_comments: Comment on the trap (besides colour and bait).
language	Languages used in the dataset (fr | en).
country	Country covered by the dataset (France).
basisOfRecord	Nature of the data record (Occurrence).
countryCode	Data are in continental France and Corsica only and are thus attributed the “FR” code.
day	Day at the end of sampling (trapping).
geodeticDatum	Spatial coordinate system used in the dataset (EPSG:4326 = WGS84).
kingdom	Animalia.
class	Insecta.
order	Coleoptera.
occurrenceRemarks	Information on the locality.
samplingProtocol	Type of trap.

## Additional information

### Description of the standardised dataset and comparison with data from other collection methods

We compared the present standardised dataset focused on collections from flight interception traps (DATASAPROX) with a dataset gathering other types of collection methods (e.g. opportunistic expert observations, citizen sciences, aerial Malaise or baited bottle traps, emergence and malaise traps, …) which is based on data available on the French SINP platform via the OpenObs web portal (INPN - Données d'observation et de suivi sur les espèces). This non-standardised dataset could not readily be obtained from the SINP website, as information on the sampling protocol and on the saproxylic nature of taxa is not directly provided. To create this dataset, we first had to extract the entire data on Coleoptera from metropolitan France and Corsica from the website, then select only saproxylic beetles, based on the French list of species (Bouget et al. 2019) and, finally, remove the part corresponding to flight interception trap samplings by using the geographic coordinates and dates from the DATASAPROX dataset. Table 4 presents summary statistics on both datasets.

Beside the sampling methods, one notable difference between the DATASAPROX and the OpenObs datasets concerns the number of data producers. The DATASAPROX dataset involves a select number of actors, firstly the French forestry agency (ONF), then research departments (e.g. INRAE EFNO), biodiversity management agencies (e.g. CEN) and professional entomologists (e.g. Benoit DODELIN), while the OpenObs dataset involves a large number of mainly non-professionals sharing their opportunistic observations (thousands), in addition to the actors also present in the DATASAPROX dataset. As a result, a much greater number of municipalities (10 times more) and geographic points (15 times more) are included in the OpenObs dataset compared to the DATASAPROX one, with also a greater number of genera and species identified, while the OpenObs dataset has only 10% more records. The DATASAPROX dataset has an average number of samples by geographic point greater than in the OpenObs dataset (7.2 vs. 2), which is because flight interception traps are usually maintained on the same location over several sampling seasons contrary to opportunistic observations and other non-standardised samplings. Another difference between the present dataset and the OpenObs one is the geographic and temporal precision, as flight interception trap samplings are more often associated with precise geographic locations and dates than opportunistic observations and non-standardised samplings. Finally, we can also see in Table 4 that data in the OpenObs dataset date back to the year 1700, although most data were accumulated after 1990, while the oldest date in the DATASAPROX dataset is 1997, since the usage of flight interaction traps started to develop from this time onwards (Nageleisen and Bouget 2009a, Brustel 2012) (see Fig. 2).

**Sampling effort in the DATASAPROX and the OpenObs datasets**

**Figure 4.**
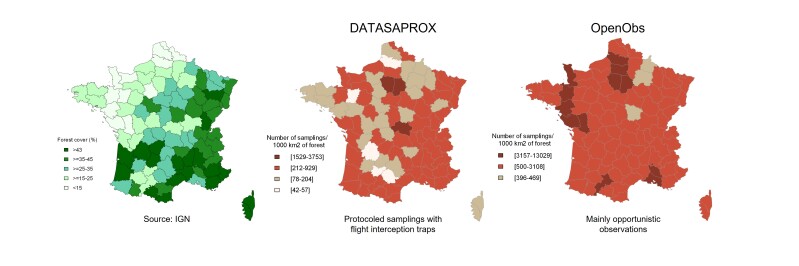
Left panel: Forest cover per county estimated by the French national forest inventory (IGN). Centre and right panels: Number of samples (collection or observation events) by unit of forest available in each county for the present DATASAPROX dataset and the other data from the OpenObs web platform.

The sampling effort is less evenly distributed across the different counties in the DATASAPROX dataset compared to the OpenObs one (Fig. 4), with clear gaps in geographic coverage in the DATASAPROX dataset. Samplings with flight interception traps are particularly rare in the following counties: Dordogne, Tarn-et-Garonne, Tarn, Mayenne and Somme, whereas, in the same counties, other samplings (including citizen-science and expert inventories) are abundant. Amongst those counties, Dordogne (and Tarn), efforts should be made to prioritise for future standardised sampling with flight interaction traps as they present a relatively high forest cover. It must also be noted that the sampling pressure is relatively low in the Nièvre, Ardennes and Marne counties for both datasets, while the Nièvre County is also relatively well forested.

**Taxonomic differences between the DATASAPROX and the OpenObs datasets**

**Figure 5.**
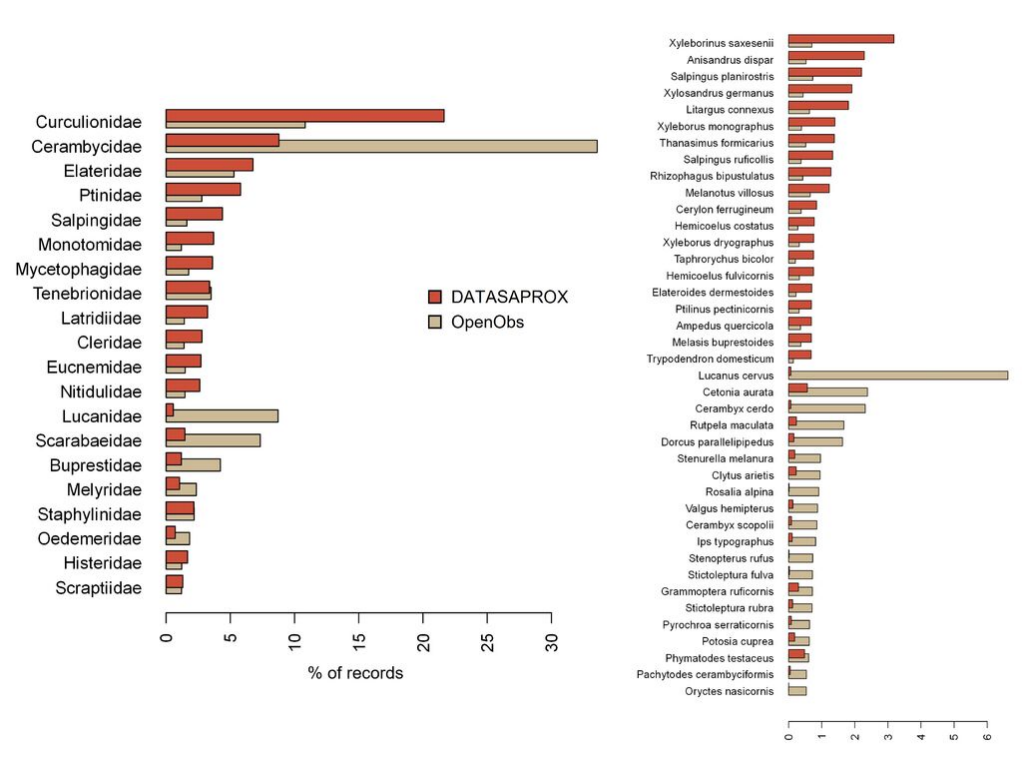
Percentage of records for the twenty most represented families (left panel) and the forty most represented species (right panel) in the present DATASAPROX dataset (brown) and for data from the OpenObs web platform (beige).

The families Cerambycidae, Lucanidae, Scarabaeidae, Buprestidae, Melyridae and Oedemeridae are largely over-represented in the OpenObs dataset compared to the DATASAPROX one (Fig. 5), while other families, well-represented in the DATASAPROX dataset, are under-represented (Curculionidae, Ptinidae, Salpingidae, Monotomidae, …). In addition, large differences in species composition can be seen between the two datasets when looking at the most represented species. The twenty most represented species in the DATASAPROX dataset are all very under-represented in the OpenObs dataset and vice versa. In the DATASAPROX dataset, those are mostly discrete and small species (e.g. Scolytinae) while, in the OpenOps dataset, we find a high prevalence of large and/or iconic species, such as *Lucanus cervus* (Linnaeus, 1758), *Cetonia aurata* (Linnaeus, 1758), *Cerambyx cerdo* Linnaeus, 1758 and *Rosalia alpina* (Linnaeus, 1758).

**Differences in species traits between the DATASAPROX and the OpenObs datasets**

The different sampling methods used in the two datasets likely explain differences in traits. The OpenObs dataset includes a greater percentage of meridional and Corsican species records than the DATASAPROX dataset (Table 5), maybe because those include species which are more targeted by opportunistic collectors and/or because there are relatively few flight interaction trap samplings in Corsica (but not in the Mediterranean region; see Fig. 4). There is also a greater proportion of rare (patrimonial), easy to identify and large species (> 20 mm) records in the OpenObs dataset, which is an expected bias from amateur opportunistic or targeted observations. Conversely, the DATASAPROX dataset includes a greater percentage of hard to identify and small species (< 3 mm) records and a lower percentage of apterous and dimorphic species records than the OpenObs dataset, which is expected in flight interaction trap passive samplings.

### Additional statistics

Table 6

Fig. 6

### Limitations and usage of the dataset

Some limitations of the dataset need to be accounted for before biodiversity estimations, conservation and community ecology analyses. Data compiled in this dataset originate from various sources with different practices in terms of surveys goals (surveys looking at rare species versus random sampling of the community), sampling design and different skills and uses in terms of sample identification and recording. In particular, some projects (field: “datasetName”) can be focused on particular families, groups or species (e.g. Curculionidae Scolytinae). To help identify these projects, we provide in the table hereafter (Table 7) the name and proportion of the most represented family, the number of families and the median number of taxa in each sample, all calculated for each project. In addition, we added an indicative field mentioning the completeness of the community (complete vs. partial). We considered a community to be partial (i.e. biased towards particular taxa or incomplete) if the main family in the project represented more than 50% of the records, the number of families was less than 10 or if the median number of taxa in each sample was less than 6. For example, the number of taxa per sample can be expected to be in the range 8–22 depending on environmental conditions, trap type or sampling duration. For each analysis question, the dataset should be reduced by selecting events comparable in terms of traps types, temporal coverage and taxonomical coverage.

## Figures and Tables

**Figure 6.**
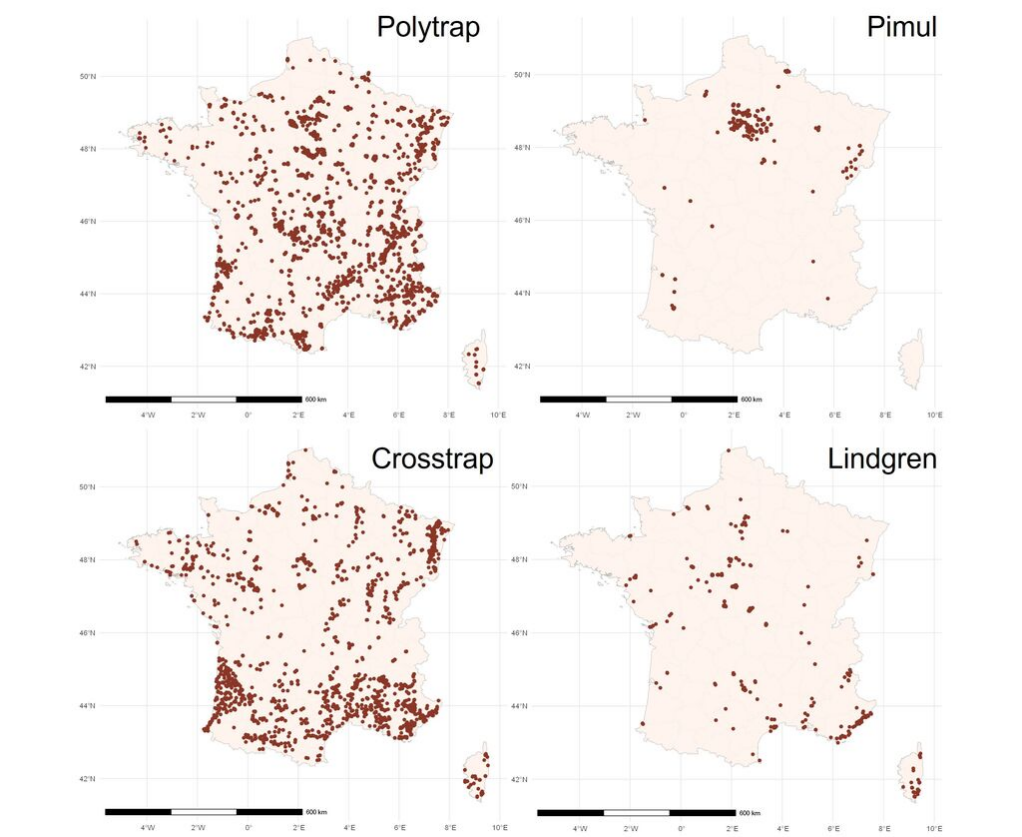
Maps of the sampling points for the four most represented trap models in the DATASAPROX dataset.

**Table 1.** List of data producers with codes in the dataset and the corresponding full names. For the agencies, the names of the main contact persons figure between parentheses.

Codes	Full names and contact persons.
ONF	Office National des Forêts (Thomas Barnouin and Fabien Soldati).
EFNO	INRAE Ecosystèmes Forestiers Nogent-sur-Vernisson (Christophe Bouget).
DODELIN	Benoit Dodelin (freelance).
PURPAN	Ecole d'Ingénieurs de Purpan (Antoine Brin, Lionel Valladares and Hervé Brustel).
OPIE	Office pour les Insectes et leur Environnement (Bruno Mériguet).
SHNAO	Société d’Histoire Naturelle Alcide-d’Orbigny (Benjamin Calmont).
URZF	INRAE Unité de Recherche de Zoologie Forestière (Alain Roques).
SEL	Société Entomologique du Limousin (Romain Chambord and Laurent Chabrol).
NMENTOMO	Nicolas Moulin (freelance).
CENOCC	Conservatoire d'Espaces Naturels Occitanie (Nicolas Gouix, Hugo Norel and Mathieu Bossaert).
CENBOURG	Conservatoire d'Espaces Naturels Bourgogne (Samuel Gomez and Vottana Tep).
CERCOPE	Coordination Entomologique de la Région Centre pour l’Organisation de Projets d’Etude (Sébastien Damoiseau).
MNHN	PatriNat - centre d’expertise et de données sur le patrimoine naturel (Julien Touroult and Olivier Delzons).
PAGES	Jacques Pages - BioDev’ mlhl.
FRAPA	Pierre Frapa (freelance).
GRETIA	GRoupe d’ÉTude des Invertébrés Armoricains (Frank Herbrecht).
GON	Groupe Ornithologique et Naturaliste agrément Hauts-de-France (Guillaume Delporte).
ADEP	Association Des Entomologistes de Picardie (Jean-Hervé Yvinec).
CCENTOMO	Christian Cocquempot (freelance).
CENPACA	Conservatoire d'Espaces Naturels PACA (Florian Buralli and Yolan Richard).
CPIELA	CPIE Loire Anjou (Olivier Durand).

**Table 2.** List of flight interaction traps considered in the dataset.

Model	Type	Colours	Baits	No. records
polytrap	cross-vane	transparent, multiple	alcohol or none (rarely terpenes, pheromones)	541,388
pimul	cross-vane	transparent	none (rarely alcohol, pheromones)	47,394
crosstrap	cross-vane	black, transparent	pheromones (rarely alcohol, terpenes)	42,513
lindgren	multifunnel	multiple	none or pheromones + alcohol + terpenes	37,954
window	single-vane	transparent	alcohol	3949
inter_OPIE_2003_2007	cross-vane	transparent	none (rarely alcohol)	1301
panel	cross-vane	multiple	pheromones + alcohol + terpenes	901
inter_OPIE_2002	cross-vane	transparent	none (rarely alcohol)	125

**Table 3.** List of the saproxylic beetle families considered in the dataset. Percentage of known species according to the French list of saproxylic Coleoptera (Bouget et al. 2019). The family Curculionidae is split between the subfamily Scolytinae and the others Curculionidae, excluding Scolytinae. Families are ordered according to Bouchard et al. (2011), Gimmel et al. (2019), Bouchard and Bousquet (2020).

Family	No. genus	No. species	% known species	No. records
Rhysodidae	1	1	100	22
Carabidae	6	17	81	1851
Hydrophilidae	4	8	89	132
Sphaeritidae	1	1	100	99
Histeridae	22	48	94	11374
Ptiliidae	9	18	33	171
Leiodidae	12	50	62	7365
Staphylinidae	96	265	60	14553
Lucanidae	6	9	82	3800
Trogidae	1	2	100	322
Scarabaeidae	12	17	81	9974
Eucinetidae	3	2	100	189
Clambidae	4	10	59	341
Scirtidae	1	1	50	124
Buprestidae	20	103	85	8046
Elmidae	1	1	25	1
Dryopidae	1	1	100	5
Cerophytidae	1	1	100	178
Eucnemidae	12	22	96	18390
Throscidae	1	2	100	3668
Elateridae	21	63	89	45865
Lycidae	8	8	100	1628
Cantharidae	2	39	51	1504
Nosodendridae	1	1	100	195
Dermestidae	8	45	63	6030
Bostrichidae	14	18	69	3008
Ptinidae	33	109	86	39248
Lymexylidae	2	2	100	5253
Phloiophilidae	1	1	100	26
Peltidae	1	2	100	117
Lophocateridae	1	1	50	12
Trogossitidae	5	5	83	3309
Thymalidae	1	1	50	1209
Rhadalidae	2	13	57	1333
Melyridae	16	49	61	7091
Cleridae	12	21	95	18955
Sphindidae	4	4	100	3874
Nitidulidae	16	60	74	17766
Monotomidae	3	22	79	25054
Phloeostichidae	1	1	100	102
Silvanidae	7	9	60	3365
Cucujidae	3	3	75	1852
Laemophloeidae	7	21	95	6807
Cryptophagidae	9	65	68	3601
Erotylidae	4	13	100	10902
Biphyllidae	3	3	100	4784
Bothrideridae	5	6	35	1900
Cerylonidae	3	7	88	10251
Alexiidae	2	2	33	14
Endomychidae	6	8	89	1718
Corylophidae	4	16	89	781
Latridiidae	12	71	67	21863
Mycetophagidae	9	21	91	24482
Ciidae	12	52	96	12932
Tetratomidae	4	7	88	2061
Melandryidae	17	29	91	10623
Zopheridae	11	20	74	10034
Mordellidae	10	29	85	3531
Tenebrionidae	37	83	86	23003
Prostomidae	1	1	100	145
Oedemeridae	7	19	83	4823
Pythidae	1	1	100	39
Pyrochroidae	2	3	75	1425
Salpingidae	6	16	94	29666
Aderidae	9	11	85	1159
Scraptiidae	4	29	67	8848
Cerambycidae	98	198	90	59575
Anthribidae	16	22	79	7167
Brentidae	2	2	100	23
Dryophthoridae	1	2	100	1104
Curculionidae Scolytinae	45	145	90	135197
Curculionidae others	35	81	72	9666

**Table 4.** Summary information on the present DATASAPROX dataset and comparison with other data from the SINP platform (OpenObs web portail).

	DATASAPROX	OpenObs
collection methods	flight interception traps	opportunistic observations, others
no. records	675525	752676
no. data producers	21	> 1000
no. identified projects	485	2016
no. counties	96	96
no. municipalities	2336	23499
no. geographic points	8969	141863
% precise points	86	51
no. samples or observations	66202	286705
years (min-median-max)	1997-2016-2025	1700-2010-2025
% precise dates	100	71
no. families	71	74
no. subfamilies	153	160
no. genera	738	795
no. species	2039	2344
% species level identif.	98	98
% abundances (vs. presences)	88	undefined

**Table 5.** Proportion of traits from the FRISBEE trait dataset (Bouget et al. 2019) in the present DATASAPROX dataset and for data from the other data from OpenObs web platform. The numbers are the percentages of records where species with the trait occur.

Trait	DATASAPROX	OpenObs
Meridional	3.9	7.3
Corsican	0.027	0.076
Rare	4	7.3
Apterous/dimorphic	1	2.1
Size < 3 mm	27	12
Size > 20 mm	0.94	15
Easy to identify	24	39
Hard to identify	22	14

**Table 6.** Summary information on the data provided by the different producers. Sampling duration and number of taxa per sample are median values.

Data producers	Trap models	No. records	No.. districts	No.. points	No. events	Years	Occurrence types	Event duration (days)	No. taxa/event
ONF	polytrap	300,267	484	1965	25,940	1997-2024	counts, presences	14	11
	crosstrap	41,878	1048	1935	5285	2013-2024	counts	11	7
EFNO	polytrap	66,687	169	739	4546	2006-2023	counts	28	13
	lindgren	21,228	34	181	1458	2016-2024	counts	29	14
	window	2944	9	50	242	2001-2001	counts	28	11
DODELIN	polytrap	60,959	161	882	7341	2002-2024	counts	15	7
PURPAN	polytrap	54,338	147	688	4743	1997-2023	counts	26	11
OPIE	pimul	42,509	139	683	4893	2004-2024	counts, presences	19	9.5
	polytrap	2754	9	28	305	2011-2024	counts, presences	21	23
	inter_OPIE_2003_2007	1301	15	32	354	2003-2007	counts	10	2
	crosstrap	181	4	5	31	2021-2021	counts	14	4
	lindgren	137	4	4	23	2021-2021	counts	20	11
	inter_OPIE_2002	125	4	13	49	2002-2002	counts	26	2
SHNAO	polytrap	31,365	92	753	3778	2004-2024	presences	19	6
URZF	lindgren	15,577	138	210	2998	2009-2025	counts, presences	21	5
	panel	765	10	11	56	2015-2024	counts, presences	17	25
	crosstrap	395	3	5	100	2015-2017	counts	8	5
SEL	polytrap	8446	75	330	1303	2001-2024	presences	18	5
NMENTOMO	pimul	4854	15	71	522	2008-2021	counts	21	11
	polytrap	253	1	4	25	2024-2024	counts	14	9
CENOCC	polytrap	3127	29	123	413	2010-2024	counts, presences	26	10
CENBOURG	polytrap	1987	2	18	211	2022-2024	counts	14	8
	window	966	2	6	110	2022-2024	counts	14	8
CERCOPE	polytrap	2617	9	53	206	2020-2024	counts	31	11
MNHN	polytrap	1438	6	51	120	2019-2022	counts	14	10
	lindgren	1012	4	38	88	2019-2021	counts	14	10
PAGES	polytrap	1780	4	38	324	2011-2019	counts	14	4
FRAPA	polytrap	1364	11	33	206	2004-2025	counts, presences	20	6
	pimul	31	2	2	4	2008-2008	counts	15	4
GRETIA	polytrap	1264	29	51	159	2014-2024	counts, presences	14	8.5
	window	39	8	8	29	2014-2014	counts	<1	8
GON	polytrap	1127	3	5	66	2022-2024	counts	14	15
ADEP	polytrap	1021	10	16	85	2023-2025	counts	12	10
CCENTOMO	polytrap	507	16	31	149	2009-2021	presences	17	3
	panel	136	5	8	36	2019-2021	presences	23	3
	crosstrap	58	4	5	18	2013-2014	presences	11	3
CENPACA	polytrap	44	10	16	21	2004-2022	counts, presences	23	2
	crosstrap	1	1	1	1	2014-2014	counts	30	1
CPIELA	polytrap	43	7	12	14	2012-2023	counts	<1	2

**Table 7.** Information on the community structure, diversity and completeness for each identified project (“datasetName” field).

ownerInstitutionCode	datasetName	Dominant family	No. families	Median no. taxa/sample	cCommunity
ADEP	Forêt du Touquet 2023	Elateridae 22%	27	7	complete
ADEP	Forêt de Crécy 2024	Latridiidae 13.9%	29	22	complete
ADEP	Coteaux Vallée Automne 2025	Monotomidae 11.2%	33	11	complete
ADEP	Forêt de Retz 2023-2024	Curculionidae 11.3%	42	10	complete
ADEP	Forêt de Coucy St-Gobain 2025	Mycetophagidae 12%	30	10	complete
CCENTOMO	sans_nom_de_projet_CCENTOMO	Cerambycidae 38.4%	32	3	partial
CENBOURG	[AXE1] Sites Axe 1 - Inventaires, amélioration et connaissance	Curculionidae 19.1%	44	6	complete
CENBOURG	[RN] Réserves naturelles - Inventaire, amélioration et connaissance	Curculionidae 14.5%	43	8	complete
CENOCC	COLEO_GABIZOS - Polytrap	Leiodidae 12.9%	32	6.5	complete
CENOCC	BLANCASTET_2014 - Polytrap	Elateridae 14.3%	12	1	partial
CENOCC	BLANCASTETCOLEO_2015 - Polytrap	Curculionidae 19%	36	3	partial
CENOCC	MOURA_2014 - Polytrap	Tenebrionidae 18.8%	11	1	partial
CENOCC	B_ABICASTARAC - Polytrap	Curculionidae 32%	25	4	partial
CENOCC	hors étude particulière - Polytrap	Curculionidae 30.5%	30	21	complete
CENOCC	ABC_2014 - Polytrap	Elateridae 18.2%	9	1	partial
CENOCC	BIOFORDIV - Polytrap	Curculionidae 11.4%	45	10	complete
CENOCC	EXP46_2015 - Polytrap	Elateridae 20.3%	20	1	partial
CENOCC	CAPLONG_2017 - Polytrap	Salpingidae 27.3%	7	1	partial
CENOCC	CAYLUS_2011 - Polytrap	Staphylinidae 17.9%	16	2.5	partial
CENOCC	A_COLEO_ARIEGE_Inventaire_Saprox_ANA - Polytrap	Curculionidae 30.4%	41	10.5	complete
CENOCC	A_NATUREIMPACT_WWF_COUNOZOULS - Polytrap	Salpingidae 21.4%	27	3	partial
CENOCC	MILITAIRES_LARZAC - Polytrap	Melyridae 37.5%	4	1	partial
CENOCC	OCVIA : Suivis et état zéro - Polytrap	Cerambycidae 17.6%	25	15.5	complete
CENOCC	A_MC_CD30_STE_CECILE_ANDORGE - Polytrap	Curculionidae 12%	37	43.5	complete
CENPACA	- Aucun programme -	Ptinidae 39.1%	9	1	partial
CENPACA	ABC du Lauzet - Ubaye	Melyridae 27.3%	14	3	partial
CERCOPE	sans_nom_de_projet_CERCOPE	Curculionidae 17.4%	53	11	complete
CPIELA	2023 - BI461 - Cléré-sur-Layon	Scarabaeidae 60%	2	2.5	partial
CPIELA	2015 - NE369 - Projet Parcs et Châteaux	Curculionidae 87.5%	2	4	partial
CPIELA	0 - Aucune	Curculionidae 70%	7	2	partial
DODELIN	PNR des Boucles de la Seine	Curculionidae 13.1%	41	7	complete
DODELIN	Dir Espaces Verts CG 78	Curculionidae 15.8%	33	7	complete
DODELIN	Biotope	Latridiidae 12.8%	53	7	complete
DODELIN	Biotope, SAFER	Curculionidae 29.2%	17	10	complete
DODELIN	CG 77	Elateridae 13.7%	47	4	partial
DODELIN	Agence des espaces verts de la Région d’Ile-de-France	Curculionidae 28.5%	41	7	complete
DODELIN	Epafrance	Elateridae 45%	23	4	partial
DODELIN	Acer Campestre	Curculionidae 25%	29	13	complete
DODELIN	PNR du Morvan	Curculionidae 18.7%	45	7	complete
DODELIN	Conservatoire d’Espaces Naturels Rhône-Alpes	Curculionidae 12.3%	55	4	partial
DODELIN	PNR de la Montagne de Reims	Curculionidae 16.5%	47	6	complete
DODELIN	Syndicat des Rivières Beaume et Drobie	Elateridae 12.7%	39	11	complete
DODELIN	RN des Gorges de la Loire	Elateridae 14.1%	49	6	complete
DODELIN	PNR des Ardennes	Curculionidae 18%	19	1	partial
DODELIN	ONF 07	Curculionidae 15.6%	53	8	complete
DODELIN	RN de l'Île de la Platière	Throscidae 10.8%	38	4	partial
DODELIN	EPTB Saône & Doubs	Curculionidae 22.3%	49	17.5	complete
DODELIN	Smiril	Latridiidae 14.6%	39	3	partial
DODELIN	ENS Pierre-Aiguille	Tenebrionidae 11.2%	35	13	complete
DODELIN	VINCI, Écosphère SE	Zopheridae 18.5%	19	6.5	complete
DODELIN	Biotope, Métropole de Lyon	Latridiidae 14.9%	35	13	complete
DODELIN	RNN des Ramières	Scraptiidae 17.8%	42	7	complete
DODELIN	DDAF 26	Cerambycidae 14.4%	35	5	partial
DODELIN	CG 26, REFORA, ONF 26	Curculionidae 24.9%	45	8	complete
DODELIN	PNR du Vercors	Curculionidae 23.4%	57	11	complete
DODELIN	CNR Compagnie Nationale du Rhône	Curculionidae 18.7%	49	9	complete
DODELIN	Organom	Curculionidae 16.9%	53	12	complete
DODELIN	ENS Les Coteaux de Saint-Roch	Curculionidae 20%	42	10	complete
DODELIN	Communauté de communes du Diois	Curculionidae 20.2%	41	12	complete
DODELIN	CG 26, DDAF 26	Curculionidae 23.4%	40	7	complete
DODELIN	Lo Parvi	Curculionidae 18%	46	7	complete
DODELIN	Commune de Rencurel	Curculionidae 27.9%	31	11	complete
DODELIN	CG 38, ONF	Curculionidae 22.9%	46	4	partial
DODELIN	CG 38	Curculionidae 18.9%	49	8	complete
DODELIN	Syndicat du Haut-Rhône	Curculionidae 21.2%	42	8	complete
DODELIN	ONF 38	Curculionidae 26.7%	47	4	partial
DODELIN	Mosaïque Environnement & EDF	Latridiidae 19.1%	22	9	complete
DODELIN	Communauté d’agglomération du Grand Dole	Curculionidae 42.2%	47	8	complete
DODELIN	Conservatoire d’espaces naturels Savoie	Curculionidae 18%	58	7	complete
DODELIN	Aucun	Staphylinidae 11.3%	43	5	partial
DODELIN	RN du Marais de Lavours	Curculionidae 17.3%	39	3	partial
DODELIN	ONF	Curculionidae 21.6%	53	8	complete
DODELIN	RNR du lac d’Aiguebelette	Latridiidae 18.8%	39	7	complete
DODELIN	RN du Pont des Pierres, LPO Rhône-Alpes	Curculionidae 23.9%	27	3	partial
DODELIN	Commune de La Motte Servolex	Curculionidae 33.2%	34	5	partial
DODELIN	RN du Lac Luitel	Curculionidae 46.1%	24	2	partial
DODELIN	PNR du Haut-Jura	Curculionidae 29.2%	48	7	complete
DODELIN	Conservatoire d’espaces naturels Savoie & Conservatoire du littoral	Curculionidae 12.7%	37	8.5	complete
DODELIN	Avenir	Curculionidae 14.2%	41	9	complete
DODELIN	PN Ecrins	Curculionidae 26.3%	44	3	partial
DODELIN	PNR du Massif des Bauges	Curculionidae 27%	36	5.5	complete
DODELIN	Communauté de communes Bas Chablais	Curculionidae 19%	27	9	complete
DODELIN	ONF 74	Curculionidae 40.7%	34	6	complete
DODELIN	Aucun: autorisation et info à ONF 73	Nitidulidae 43.5%	9	2	partial
DODELIN	ONF 73	Curculionidae 38.4%	21	4.5	partial
DODELIN	ONCFS Gap	Curculionidae 35%	42	6	complete
DODELIN	RN du Plan de Tuéda	Curculionidae 31.8%	28	6	complete
DODELIN	INSECTA	Latridiidae 13.5%	36	8	complete
DODELIN	PN de la Vanoise	Curculionidae 43.8%	29	2	partial
EFNO	DEPRE	Curculionidae 99.8%	2	3	partial
EFNO	CLIMTREE	Curculionidae 20.2%	48	13	complete
EFNO	CANOPEE_EFNO	Cerambycidae 14.8%	51	14	complete
EFNO	RBI-RBD Rambouillet Cemagref	Curculionidae 24.1%	50	12	complete
EFNO	AL	Curculionidae 17%	50	14	complete
EFNO	BUCHE L	Buprestidae 20.5%	46	17	complete
EFNO	THERA	Curculionidae 15.8%	44	18	complete
EFNO	FORGECO	Curculionidae 13.8%	42	14	complete
EFNO	REGIX	Curculionidae 22.6%	31	8.5	complete
EFNO	JYBORLEANS	Curculionidae 24.9%	40	13	complete
EFNO	BRIE	Monotomidae 17%	31	11	complete
EFNO	INVBARRES	Curculionidae 14.9%	35	12	complete
EFNO	MANIBARRES	Curculionidae 12.6%	44	28.5	complete
EFNO	ColLindN	Curculionidae 16.4%	18	7	complete
EFNO	DISTR	Curculionidae 22.8%	47	17	complete
EFNO	PERCEL	Curculionidae 17.3%	48	13	complete
EFNO	GNB BDP	Curculionidae 21.5%	26	9.5	complete
EFNO	GNB HT	Nitidulidae 17.5%	33	14	complete
EFNO	GNB CL	Elateridae 15%	32	9	complete
EFNO	JANSSEN	Curculionidae 29.9%	51	11	complete
EFNO	HCJ	Curculionidae 18.7%	44	15	complete
EFNO	HAYE	Curculionidae 15.6%	43	12	complete
EFNO	GNB BC	Salpingidae 14.6%	28	4	partial
EFNO, ONF	GNB Auberive (52)	Curculionidae 17.9%	35	9	complete
FRAPA	Ripisylves Luberon-Lure	Curculionidae 16.8%	39	6.5	complete
FRAPA	Etude post-incendie La Thomassine	Curculionidae 18.2%	29	6	complete
FRAPA	Animation N2000-2024	Curculionidae 35.5%	13	9	complete
FRAPA	Projet tutoré IUT Digne	Curculionidae 24.1%	16	3	partial
FRAPA	FD des Pénitents 2011	Melyridae 14%	24	3	partial
FRAPA	Vallon de Rome	Curculionidae 19.6%	23	9	complete
GON	programme d’activités milieu forestier (sous-action SAPROX)	Curculionidae 22.4%	46	15	complete
GRETIA	Inventaire des invertébrés de la Corniche de Pail par piégeages aériens (53) – 2023	Elateridae 14.5%	20	7	complete
GRETIA	Etude des invertébrés de la RNR Etang et boisement de Joreau (49) (action SE13) - 2017	Salpingidae 12.6%	30	8.5	complete
GRETIA	Inventaire des coléoptères saproxyliques de deux ENS du Calvados (14) - 2023	Salpingidae 18.8%	16	2	partial
GRETIA	Inventaire des invertébrés de l'ENS des Roches d'Oëtre (61) - 2021	Curculionidae 31.1%	20	7.5	complete
GRETIA	Inventaire non protocolé des invertébrés du Bois de Bretel (Couvains, F.50) - 2021	Curculionidae 42.3%	18	12	complete
GRETIA	Données personnelles Pierre Devogel	Curculionidae 45.5%	5	2.5	partial
GRETIA	Données opportunistes - Formation ABB du 17-18/06/2021 - Paimpont (35) - 2021	Tenebrionidae 33.3%	3	3	partial
GRETIA	Inventaire des coléoptères saproxyliques par piège polytrap de la RBI de la Butte de Malvran (56) - 2024	Curculionidae 35.6%	25	15	complete
GRETIA	Inventaire des coléoptères saproxylophages de la commune de Saint-Agathon (22) - 2022	Curculionidae 35.3%	8	8.5	partial
GRETIA	Inventaire des invertébrés de l'ENS de la vallée du Blavet - 22 - 2016	Curculionidae 36.4%	5	3	partial
GRETIA	Inventaire des invertébrés de l'ENS de la Vallée du Léguer (22) - 2015	Curculionidae 52.3%	8	8	partial
GRETIA	Inventaire de l'ENS de la Forêt départementale de Coat-Mez (29) - 2022/2023	Curculionidae 32.1%	19	12	complete
GRETIA	Inventaire des coléoptères saproxyliques de la RNR du coteau et prairies des Caforts (72) - 2020	Ptinidae 19.4%	21	5	partial
GRETIA	Étude Gretia : programme forêts PDL travaux préliminaires - Invertébrés 2014	Cerambycidae 66.7%	3	3	partial
GRETIA	Inventaire des coléoptères saproxyliques pour l'ABC de Caux-Seine agglo (76) - 2022	Curculionidae 32.1%	21	11	complete
GRETIA	Données personnelles des bénévoles du Gretia	Curculionidae 42.9%	8	14	partial
MNHN	Patrinat - Brunoy	Curculionidae 13%	30	69	complete
MNHN	Patrinat - Sainte-Foy-Tarentaise	Cerambycidae 19%	18	13	complete
MNHN	La Planète Revisitée	Curculionidae 18.4%	42	10	complete
NMENTOMO	100Vergers 2009	Ptinidae 21.8%	22	4.5	partial
NMENTOMO	PNRBSN 2009	Ptinidae 23.1%	23	4	partial
NMENTOMO	Barentin Bois Bénard 2018	Ptinidae 13.2%	31	12	complete
NMENTOMO	Barentin Bois Bénard 2021	Ptinidae 14.1%	28	10.5	complete
NMENTOMO	MAP 2018	Latridiidae 17.1%	25	9	complete
NMENTOMO	Perray en Yvelines 2019	Cerambycidae 10.7%	33	11	complete
NMENTOMO	Marines 2011	Ptinidae 9.4%	41	9	complete
NMENTOMO	Hautil 2010	Elateridae 12.7%	34	6	complete
NMENTOMO	Hautil 2011	Curculionidae 16.5%	36	11	complete
NMENTOMO	Hautil 2012	Curculionidae 15.3%	43	11	complete
NMENTOMO	RNR Mesnil le Roi 2008	Ptinidae 25%	16	4	partial
NMENTOMO	Ollainville 2021	Cerambycidae 18%	28	12.5	complete
NMENTOMO	Boulogne 2024	Curculionidae 16.7%	23	6.5	complete
NMENTOMO	Vincennes 2024	Curculionidae 13.4%	24	9	complete
NMENTOMO	ANDRA 2009	Elateridae 10.3%	22	6	complete
NMENTOMO	ANDRA 2014	Latridiidae 18.2%	28	5	partial
ONF	Piègeage Monochamus	Curculionidae 30%	47	7	complete
ONF	RB Ecouves	Curculionidae 30.7%	32	12	complete
ONF	ETIENNE Sébastien	Curculionidae 28.9%	49	10	complete
ONF	Bel Air Sauvignac - LGV ouest (16)	Curculionidae 43.4%	26	6	complete
ONF	Monochamus_2024	Curculionidae 29.3%	42	6	complete
ONF	N2000 Monts d'Eraines (14)	Curculionidae 33.9%	33	10	complete
ONF	Puit d'Enfer (79) - Projet ENS	Curculionidae 31.4%	34	15.5	complete
ONF	Bois de Touvérac - LGV ouest (16)	Curculionidae 27.2%	34	15	complete
ONF	VAN MEER Cyrille	Curculionidae 10.8%	58	7.5	complete
ONF	GNB Chizé	Curculionidae 10.5%	38	10	complete
ONF	Bussac Forêt - LGV ouest (17)	Curculionidae 37.1%	36	12	complete
ONF	RNR Bocage des Antonins (79)	Curculionidae 24.7%	43	18	complete
ONF	FERCHAUD Laurent	Curculionidae 18.9%	35	6	complete
ONF	Andaine FD	Curculionidae 42.1%	34	9	complete
ONF	Marais de Galuchet (79) - Projet RN	Curculionidae 28.2%	29	11.5	complete
ONF	Bordeaux Parc (33)	Curculionidae 27.7%	37	9	complete
ONF	VELLE Laurent	Curculionidae 20.5%	60	9	complete
ONF	FD de Cerisy	Curculionidae 36.4%	34	10	complete
ONF	VAYSSIE Jean-Philippe	Cerambycidae 16.7%	30	6	complete
ONF	FD Lège et Garonne (33)	Curculionidae 36.6%	35	12	complete
ONF	MICAS Lilian	Curculionidae 15.7%	60	6	complete
ONF	FD de la Teste de Buch (33)	Curculionidae 37.1%	41	12	complete
ONF	RBD Pointe d'Arçay (85)	Curculionidae 45.6%	26	9	complete
ONF	RBI de la Mailloueyre (40)	Curculionidae 38.2%	30	10	complete
ONF	JEANNEAU Anthony	Curculionidae 26.5%	42	7	complete
ONF	Landevennec îlot vieillissement (29)	Curculionidae 42.1%	24	6	complete
ONF	RBI Landevenec (29)	Curculionidae 34.4%	25	6.5	complete
ONF	Bois de Pioussay - LGV ouest (79)	Curculionidae 29.2%	34	14	complete
ONF	Bocage de Pliboux - LGV ouest (16)	Curculionidae 28.9%	32	15	complete
ONF	Poitou-Charentes Nature LGV Sud-Ouest	Curculionidae 31.6%	38	14	complete
ONF	St-Amand-de-Boixe - LGV ouest (16)	Curculionidae 30%	31	10.5	complete
ONF	RNR Parigné-l'Eveque (72)	Curculionidae 35.5%	30	9.5	complete
ONF	FD Bercé	Curculionidae 34.2%	36	13	complete
ONF	RB Vallon de Maupas en FD Chinon (37)	Curculionidae 37.9%	38	8.5	complete
ONF	Remijoux - LGV ouest (86)	Curculionidae 26.9%	31	19.5	complete
ONF	FD de Montreich (31)	Curculionidae 38.8%	35	9	complete
ONF	RBI de la Froux (28)	Curculionidae 31.7%	33	14	complete
ONF	GNB Bethmal (09)	Curculionidae 23.7%	30	6	complete
ONF	Merlimont, RBD Côte d'Opale	Curculionidae 28.1%	23	8	complete
ONF	GNB Rambouillet	Curculionidae 13.9%	36	9	complete
ONF	Domaine Présidentiel Rambouillet	Curculionidae 36.4%	31	13	complete
ONF	GNB Frau (11)	Curculionidae 19.5%	33	7	complete
ONF	RBI Gorges de la Frau (11)	Salpingidae 22.4%	27	4	partial
ONF	Bernard Boutte DSF	Curculionidae 48.3%	24	3	partial
ONF	RBI du Rosier	Curculionidae 27.4%	33	13.5	complete
ONF	Site des Camporells (66)	Curculionidae 42.8%	21	6	complete
ONF	TM 71 Réserne naturelle	Curculionidae 22.4%	34	7	complete
ONF	ENS 92	Curculionidae 44.3%	39	5	partial
ONF	Test Galloprotect Quillan (11)	Curculionidae 28.2%	15	5	partial
ONF	ROSE Olivier	Curculionidae 20.6%	60	11	complete
ONF	Gorges de la Rhue, Dordogne et Allier	Curculionidae 26.3%	47	12	complete
ONF	GNB Verrières	Curculionidae 14.7%	32	12	complete
ONF	Paris, Bois de Boulogne et Bois de Vincennes	Curculionidae 27.1%	50	7	complete
ONF	RN Nyer (66)	Curculionidae 38.5%	21	5	partial
ONF	RBI Verrières (92)	Monotomidae 13.4%	28	4	partial
ONF	RBD de la Cailleuse FD Montmorency	Salpingidae 16%	26	7	complete
ONF	Terrain militaire de La Courtine (23)	Curculionidae 20.8%	33	22	complete
ONF	RN Jujols (66)	Curculionidae 40.6%	30	6	complete
ONF	Forêt de Tronçais	Curculionidae 25%	48	10	complete
ONF	RN Mantet (66)	Ptinidae 26.4%	25	2	partial
ONF	Inventaire JUJOLS	Curculionidae 22.4%	33	13.5	complete
ONF	FD d'Allogny (18) - îlots	Curculionidae 30.2%	41	14.5	complete
ONF	Forêt Départemental Montagne Noire (11)	Curculionidae 32.6%	40	18	complete
ONF	RN Py 2012	Curculionidae 33.7%	25	5	partial
ONF	Natura 2000 - Pin de Salzmann (66)	Curculionidae 34.6%	25	9	complete
ONF	Py (66) Inventaire des Coléoptères forestiers	Curculionidae 22.2%	26	13.5	complete
ONF	RBD du Canigou (66)	Curculionidae 44.8%	30	10.5	complete
ONF	GNB Fontainebleau (77)	Curculionidae 34.6%	31	16.5	complete
ONF	LATHUILLERE Laurent	Curculionidae 19.3%	57	10	complete
ONF	RBI de Nantigny (03)	Curculionidae 21.1%	43	19	complete
ONF	RBI Futaie Colbert (03)	Curculionidae 25.3%	40	13.5	complete
ONF	RBD des Grands-Monts (60)	Curculionidae 20%	42	30	complete
ONF	Massif du Caroux	Cerambycidae 29.1%	12	2	partial
ONF	FD Compiègne - RBI des Beaux-Monts (60)	Curculionidae 25.7%	46	15	complete
ONF	Narbonne - Puit de Carbone	Cerambycidae 27.5%	10	2	partial
ONF	RB Fontfroide	Curculionidae 48.4%	13	5	partial
ONF	RB Pas de la Lauze (34)	Curculionidae 18.7%	33	13	complete
ONF	FAVRE Eric	Curculionidae 22%	31	7	complete
ONF	FD des Colettes (03)	Curculionidae 26.4%	37	12	complete
ONF	RBI Retz (02)	Curculionidae 32.6%	41	18	complete
ONF	PNC Hourtous - FS La Malène (48)	Curculionidae 42.4%	29	3	partial
ONF	RN Mardelles de Prémery (58)	Curculionidae 31.1%	37	12.5	complete
ONF	Bout de Côte - PNC (48)	Curculionidae 40.4%	21	6.5	complete
ONF	RBI de Jaillac (10)	Curculionidae 24.4%	35	12	complete
ONF	Bramabiau - PNC (30)	Curculionidae 28.2%	42	11	complete
ONF	Raismes, RBI de Cernay	Elateridae 22.8%	24	6	complete
ONF	Ripisylve Montbrun - PNC (48)	Curculionidae 20.4%	35	11.5	complete
ONF	GNB Aigoual (48)	Ptinidae 11.2%	40	12.5	complete
ONF	RBI Lozère	Curculionidae 13.8%	39	7	complete
ONF	Puechabon - Puit de Carbone	Curculionidae 32.8%	10	4	partial
ONF	RBM Valat de l'Hort de Dieu	Curculionidae 27.9%	41	7	complete
ONF	Aigoual - Cascade d'Orgon (30) - PNC	Curculionidae 19.8%	38	9	complete
ONF	Bois du SAPET - Parc National des Cévennes (48)	Curculionidae 32.6%	37	9	complete
ONF	Baume Dolente - PNC (48)	Curculionidae 33.6%	36	7	complete
ONF	Forêt de Bézuc - Le Pompidou - PNC (48)	Curculionidae 18.3%	25	21	complete
ONF	Nord Forez Natura 2000	Curculionidae 29.5%	37	14	complete
ONF	Ramponenche - PNC (48)	Curculionidae 29.5%	42	8	complete
ONF	Ubac Can Noire - PNC (48)	Curculionidae 33%	21	6	complete
ONF	RN Meandre des Germains (03)	Curculionidae 36.7%	31	6	complete
ONF	Vergougnous - PNC (48)	Curculionidae 27.5%	34	11.5	complete
ONF	Roc de Monts - PNC (48)	Curculionidae 26.2%	33	10.5	complete
ONF	Catusse - PNC (48)	Curculionidae 27.3%	39	11	complete
ONF	Frênaie Mogour - PNC (48)	Curculionidae 23.8%	17	11	complete
ONF	Mont Lozère - Bois noir (48) - PNC	Curculionidae 23.8%	37	5.5	complete
ONF	GNB Mt Lozère (48)	Curculionidae 17.3%	29	5	partial
ONF	Chambonnet - PNC (48)	Curculionidae 24.7%	36	8	complete
ONF	Pourcharesses - PNC (48)	Curculionidae 24.1%	23	14.5	complete
ONF	FD de Malmontet (30)	Curculionidae 25.3%	24	10	complete
ONF	Forêt de Clerguemort - PNC (48)	Curculionidae 22.8%	18	10	complete
ONF	Le Planet - PNC (30)	Curculionidae 28.9%	30	6	complete
ONF	Tour d'Olivon - PNC (30)	Curculionidae 23.6%	21	10.5	complete
ONF	FD Chambons	Curculionidae 24.5%	36	8	complete
ONF	GNB Anost	Curculionidae 16%	25	7.5	complete
ONF	RBI Vernay (71) FD Anost 2014-2016	Curculionidae 33.1%	32	10	complete
ONF	RBI Sources de l'Ardèche (07)	Curculionidae 18.9%	33	7	complete
ONF	RBD Gorges de la Canche (71)	Curculionidae 23.4%	44	21	complete
ONF	RBI de l'Artoise FD saint-Michel (02)	Curculionidae 35.7%	32	14	complete
ONF	FC de Clarensac (30)	Curculionidae 38.2%	14	3.5	partial
ONF	RBM FD Vauhalaise (51)	Cerambycidae 11.6%	34	5	partial
ONF	RN Gorges du Gardon	Curculionidae 31.4%	30	5	partial
ONF	RB Bois Sauvage (07)	Curculionidae 31.5%	32	7	complete
ONF	Inventaire vallée du FURAN	Curculionidae 44.2%	20	6	complete
ONF	FD de Valbonne (30)	Curculionidae 23%	38	18	complete
ONF	RBD Marais du Gué d'Hossus (08)	Curculionidae 34.6%	33	13	complete
ONF	RBI Bois du Ruère (21)	Curculionidae 35.7%	33	11	complete
ONF	GNB Chatillon 2013	Curculionidae 16.8%	35	10	complete
ONF	RBI Plateau Combe Noire (21)	Curculionidae 30.6%	37	11	complete
ONF	AMBOISE Paul	Curculionidae 24.7%	52	13	complete
ONF	Forêt de Montdieu (08)	Curculionidae 19.8%	30	7.5	complete
ONF	RN Combe Lavaux	Curculionidae 32.5%	40	10	complete
ONF	Trois-Fontaines - Puit de Carbone	Curculionidae 28%	30	5	partial
ONF	MILLARAKIS Philippe	Cerambycidae 11.1%	40	9	complete
ONF	GNB Citeaux	Curculionidae 22.2%	37	17	complete
ONF	RBI Bois du Roncés (52)	Curculionidae 26.9%	35	12	complete
ONF	Auberive RBI Bois des Roncés	Curculionidae 42.5%	23	10.5	complete
ONF	RN Chalmessin	Curculionidae 25.2%	35	10	complete
ONF	GNB Ventoux	Curculionidae 22.4%	31	8	complete
ONF	Bresse jurassienne	Curculionidae 23.4%	32	6	complete
ONF	RBD d'Orquevaux (52)	Curculionidae 35.7%	32	7	complete
ONF	RBI Vercors	Curculionidae 21.9%	40	8	complete
ONF	FC de Lagarde d'Apt (84)	Curculionidae 26.4%	35	16	complete
ONF	GNB Engins	Curculionidae 37%	24	5	partial
ONF	PNR Sainte-Baume (13-83)	Curculionidae 22.6%	40	14	complete
ONF	GNB Lure	Ptinidae 22.9%	30	10	complete
ONF	MEGRAT Raphael	Curculionidae 32.8%	43	6	complete
ONF	ITER - Saint-Vincent-sur-Jabron (04)	Curculionidae 25.5%	39	12	complete
ONF	Natura 2000 Hautes Alpes	Curculionidae 19%	36	7	complete
ONF	Forêt de Cadarache (13)	Curculionidae 22%	39	11	complete
ONF	RB Mallissard et RB Combe d'If (38)	Curculionidae 24.3%	34	7	complete
ONF	BROCHIER Simon	Curculionidae 28.8%	46	6	complete
ONF	DSF - Suivi X. crassiusculus	Curculionidae 39.3%	34	4	partial
ONF	RBI Aulp du Seuil - Chartreuse (38)	Curculionidae 44.5%	28	8.5	complete
ONF	ITER - Mazaugues (83)	Curculionidae 40.3%	32	10	complete
ONF	Jura RN Haute Chaine	Elateridae 27.9%	16	3	partial
ONF	Pierre KLEIN	Curculionidae 21.4%	43	10	complete
ONF	Grand Vallon - Puit de Carbone	Cerambycidae 35.9%	15	9	complete
ONF	EDF Combe Madame	Curculionidae 36.7%	26	5	partial
ONF	FD du Boscodon (05)	Curculionidae 27.4%	34	8	complete
ONF	RBI d'Aiguines (83)	Curculionidae 24.3%	33	10	complete
ONF	Maures 2009 Post-incendie (83)	Elateridae 17%	26	4	partial
ONF	Prison de Draguignan (83)	Curculionidae 45.1%	19	10	complete
ONF	RNR Tourbière des Saisies (73)	Curculionidae 41.4%	33	6.5	complete
ONF	RBI Hemilly (57)	Curculionidae 27.2%	31	9	complete
ONF	GNB Parroy	Curculionidae 15.5%	30	11	complete
ONF	PNR Queyras (05)	Curculionidae 19.3%	37	4	partial
ONF	Combe Queyras - PNR (05)	Curculionidae 31.4%	25	7	complete
ONF	FD St-Antoine (70) - Chiro-Entomo	Curculionidae 37.4%	42	15	complete
ONF	Rommersberg	Curculionidae 17.1%	34	9	complete
ONF	FUCHS Ludovic	Curculionidae 25.6%	58	17	complete
ONF	RNN Haut Villaroger (73)	Curculionidae 33.6%	29	7	complete
ONF	FC Storckensohn	Curculionidae 34.7%	21	12	complete
ONF	RBM du Cheiron (06)	Curculionidae 21.1%	40	18	complete
ONF	FD de l'île Sainte-Marguerite (06)	Curculionidae 39.9%	25	6	complete
ONF	FD Guebwiller	Curculionidae 38.8%	16	9	complete
ONF	MATT Francis	Elateridae 15%	37	37	complete
ONF	GODINAT Gilles	Curculionidae 26.9%	28	6	complete
ONF	RBI Tête d'Alpes (06)	Salpingidae 17.3%	23	6.5	complete
ONF	RBI de Rothenbruch (57)	Curculionidae 22.6%	47	16	complete
ONF	FD de Krittwald (67) - Arcos	Curculionidae 20.7%	42	20	complete
ONF	Foret du Fango, RBI Malazanca - site 2 - Corse	Curculionidae 31.5%	30	14	complete
ONF	MADARY Julien	Curculionidae 24.6%	33	12	complete
ONF	Corse - Melu Moltifao	Curculionidae 32.7%	35	12.5	complete
ONF	FT de Punteniellu (20)	Curculionidae 24.8%	40	20	complete
ONF	FT de Vizzavone (2B)	Curculionidae 17.3%	32	18	complete
ONF, EFNO	Thèse Parmain	Curculionidae 12.8%	54	21	complete
ONF, EFNO	GNB Ventron (88)	Salpingidae 32.6%	21	3	partial
ONF, GRETIA	Forêts Sud-Bretagne pour le GRETIA	Curculionidae 37.4%	33	12	complete
ONF, GRETIA	Forêts Nord Bretagne pour le GRETIA 2014-2016	Curculionidae 34.7%	32	8	complete
ONF, OPIE	Suivi de la recolonisation des plantations post-tempête par l’entomofaune forestière en Forêt Domaniale du Mans (Seine et Marne) (2011-2012-2013)	Elateridae 30.2%	31	3	partial
OPIE	Etude des coléoptères saproxyliques des affluents de la Douze et du Ciron	Curculionidae 28.1%	45	4	partial
OPIE	Inventaire entomologique (Coléoptères saproxyliques et coprophages) du site Natura 2000 des coteaux du Tursan.	Mycetophagidae 16%	30	4	partial
OPIE	Les vieux arbres, un patrimoine d’avenir pour la Nouvelle-Aquitaine	Curculionidae 23.3%	37	9	complete
OPIE	Etude des Coléoptères saproxyliques d’un site engagée en libre-évolution en contexte Natura 2000 : FR7200721 - Vallées de la Grande et de la Petite Leyre	Curculionidae 34.9%	51	7	complete
OPIE	Etudes Coléoptères saproxyliques ilot de vieillisement PNRB vosges	Curculionidae 19.8%	43	11	complete
OPIE	Inventaire entomologique de l'ENS de Saint-Georges-sur-Eure	Curculionidae 10.9%	28	9.5	complete
OPIE	Étude des coléoptères saproxyliques en vallée de l’Orge	Curculionidae 16.3%	54	12	complete
OPIE	Inventaire entomologique de la carrière de Vigny	Cerambycidae 17.3%	16	2	partial
OPIE	Evaluation des enjeux coléoptères du château de Dampierre en Yveline	Curculionidae 13.8%	40	20.5	complete
OPIE	Inventaire entomologique d’un Espace Naturel Sensible du Val d’Oise : La butte de Rosne	Curculionidae 17.8%	23	5	partial
OPIE	Inventaire des Coléoptères de la RNN de Saint-Quentin	Ptinidae 9.7%	37	19	complete
OPIE	Etat initial Boucle de Chanteloup	Curculionidae 30.5%	18	3	partial
OPIE	Inventaire entomologique de l'E.N.S. Marais du Rabuais	Eucnemidae 33.3%	4	3	partial
OPIE	Inventaires entomologiques (Orthoptères, Odonates, Coléoptères et Lépidoptères rhopalocères) de l’Étang Neuf de Saclay	Elateridae 21.3%	18	6.5	complete
OPIE	Inventaire entomologique d’une forêt départementale du Val d’Oise : Le bois de la Tour du Lay	Curculionidae 24.4%	32	10	complete
OPIE	Inventaire entomologique du Domaine régional des Buttes du Parisis	Ptinidae 21.6%	32	5	partial
OPIE	Suivi d'aménagement de l'autodrome de Linas-Monthléry	Curculionidae 19.2%	44	14	complete
OPIE	Inventaire des Coléoptères aquatiques et terrestres de la RNR du Bassin de la Bièvre	Ptinidae 33.3%	14	3	partial
OPIE	Suivi de la diversité entomologique en forêt de Fontainebleau	Tenebrionidae 14.7%	38	2	partial
OPIE	Inventaire entomologique de l'espace Condroyer (Saint-Denis)	Ptinidae 37.3%	10	5	partial
OPIE	inventaire-diagnostic entomologique dans le cadre d’une démarche Oasis-nature (2011-2013)	Ptinidae 28.6%	13	2	partial
OPIE	Suivi de la composition des coléoptères saproxyliques des parcs départementaux de Seine-Saint-Denis 2008-2010	Ptinidae 22%	38	4	partial
OPIE	Atlas dynamique de la biodiversité de Seine-et-Marne	Curculionidae 13.1%	54	7	complete
OPIE	Suivi post-incendie en Forêt Domaniale de Sénart	Eucnemidae 18.9%	17	3	partial
OPIE	Inventaire entomologique de la Forêt Régionale de Gros-bois	Dermestidae 27.1%	15	4	partial
OPIE	Inventaire entomologique de la forêt régionale de Bréviande (77) - Lépidoptères – coléoptères – Odonates,	Curculionidae 15.2%	43	14	complete
OPIE	Parcs départementaux de Seine-Saint-Denis (Parc de la Courneuve, Parc du Sausset,Parc de la Bergère, Parc de la Fosse Maussoin), Inventaire Entomologique	Ptinidae 21.6%	23	3	partial
OPIE	Coléoptères saproxyliques du parc forestier de la poudrerie (CG93)	Ptinidae 13.8%	52	8	complete
OPIE	Inventaire entomologique du Parc de la Poudrerie Nationale de Sevran	Histeridae 13.3%	18	1	partial
OPIE	Inventaire entomologique de la Réserve naturelle de l’étang du Follet (Seine-et-Marne)	Eucnemidae 25%	13	6	complete
OPIE	Inventaire entomologique de la réserve naturelle régionale des Iles de Chelles (Lépidoptères, othoptères, odonates, coléoptères)	Ptinidae 16.4%	46	10	complete
OPIE	Inventaire entomologique des Réserves Biologiques Dirigées de la Forêt Domaniale de Fontainebleau	Elateridae 33.3%	19	2	partial
OPIE	Observations ponctuelles	Tenebrionidae 16.3%	27	1	partial
OPIE	Diagnostic écologique du Bois de Brou-inventaire des Coléoptères saproxyliques et état de la population du Grand Capricorne	Curculionidae 19%	44	11	complete
OPIE	Inventaires éclairs IDF	Curculionidae 20.7%	16	29	complete
OPIE	Recherche du Grand Capricorne (Cerambyx cerdo) en Forêt de Vaires-sur-Marnes	Curculionidae 14.2%	26	3	partial
OPIE	ENS-77 Bois de la Rochette - Coléoptères saproxyliques	Curculionidae 15.6%	33	4	partial
OPIE	Inventaire entomologique du parc municipal Faucigny-Lucinge - Melun (Seine-et-Marne)	Eucnemidae 16.3%	21	5	partial
OPIE	Inventaire entomologique de la Forêt Régionale de Ferrières-en-Brie	Eucnemidae 21.4%	13	2	partial
OPIE	Parc de livry-ENS77	Curculionidae 15.6%	19	9	complete
OPIE	ABC-Bois-le-roi	Curculionidae 21.4%	34	11	complete
OPIE	ABC-Vaux-le-Pénil	Histeridae 11.9%	22	9	complete
OPIE	Coléoptères carabidae et saproxylique de l'ENS de la carrière de l'enfer 77	Curculionidae 20%	32	12	complete
OPIE	Ferme de la vieille écluse (siège du CEN IDF) - Coléoptères saproxyliques	Ptinidae 30.4%	28	7	complete
OPIE	Coléoptères carabidae et saproxylique de l'ENS de Voulangis 77	Curculionidae 14%	35	12	complete
OPIE	Inventaire entomologique du Domaine Régional du Grand-Voyeux	Ptinidae 12.8%	38	7	complete
OPIE	Suivi coléoptères casiers innondables de Chatenay	Curculionidae 13.9%	36	17	complete
OPIE	Inventaire des coléoptères saproxyliques de quatre sites boisés gérés par le Conservatoire d’espaces naturels de Bourgogne en Puisaye (89)	Curculionidae 16.9%	44	13	complete
OPIE	Relevé entomologique en Forêt Domaniale de Chenoise (77)	Eucnemidae 16.6%	28	22.5	complete
OPIE	coléoptères saproxyliques du bassin de Cerilly	Curculionidae 22.4%	27	5	partial
OPIE	Inventaire entomologique de la Réserve naturelle nationale du Bois du Parc	Curculionidae 28.4%	33	6	complete
OPIE	Inventaire des coléoptères de la RNN de Vesles-et-Caumont 2018	Elateridae 15.1%	32	7	complete
OPIE	Recherche des coléoptères visés par la directive « Habitats » dans le périmètre du site Natura 2000 « FORÊTS, BOIS, ÉTANGS ET BOCAGE HERBAGER DE LA FAGNE ET DU PLATEAU D’ANOR » (59)	Cerambycidae 28%	13	1.5	partial
OPIE	inventaire des coléoptères du bois de Mervant (71)	Curculionidae 19.5%	27	10	complete
OPIE	Coléoptères saproxylique de la reculée d'Arbois	Curculionidae 29.1%	29	23	complete
OPIE	Etude Ori Franche Comté reculée d'arbois 2016-2017	Curculionidae 23.9%	31	27	complete
OPIE	Etude Ori Franche Comté 2018- Vallée du lanterne et de la saone (Natura 2000)	Curculionidae 25.6%	31	24	complete
OPIE	Etudes des coléoptères saproxyliques de la RNN du Frankenthal-Missheimle	Curculionidae 28.8%	43	9.5	complete
OPIE, SHNAO, FRAPA		Curculionidae 20.2%	59	4	partial
PAGES	Caroux-Espinouse	Cerambycidae 14.5%	46	4	partial
PAGES	RNN Estagnol	Scarabaeidae 12.9%	45	4	partial
PURPAN	VIEIL FOR	Salpingidae 11.3%	51	9	complete
PURPAN	MONOCHAMUS	Cerambycidae 47.1%	20	5	partial
PURPAN	TRANZFOR	Elateridae 26.4%	44	8	complete
PURPAN	REINA	Curculionidae 19.1%	48	11	complete
PURPAN	THES'GLENN	Ptinidae 12.8%	49	5	partial
PURPAN	REBISCLOU	Elateridae 8.4%	45	5	partial
PURPAN	SUBGASC	Curculionidae 17%	37	20	complete
PURPAN	RIOUMAJOU	Cerambycidae 12.4%	42	8	complete
PURPAN	HECHES	Curculionidae 23.4%	36	12	complete
PURPAN	CANOPEE_PURPAN	Curculionidae 19.7%	32	3	partial
PURPAN	SAUVETERRE	Cerambycidae 11.3%	35	33	complete
PURPAN	CONECTFOR	Curculionidae 22.1%	52	10	complete
PURPAN	COMMINGES	Curculionidae 18.8%	37	12	complete
PURPAN	N2000	Salpingidae 17.9%	20	10	complete
PURPAN	DISTRAFOR	Curculionidae 8.9%	53	29	complete
PURPAN	RAMIER	Curculionidae 18%	21	8.5	complete
PURPAN	DOUMERC	Cerambycidae 20%	9	2	partial
PURPAN	THESEHB	Tenebrionidae 16.7%	20	7	complete
PURPAN	GIROUSSENS	Erotylidae 25%	11	2	partial
PURPAN	ORLU	Curculionidae 13.1%	45	9	complete
PURPAN	COMPSYLV	Curculionidae 34.2%	17	3	partial
PURPAN	MONTNOIRE	Elateridae 24.3%	12	4	partial
PURPAN	RIALSESSE	Oedemeridae 29.4%	10	4.5	partial
PURPAN	AVEYRON	Ptinidae 15.5%	39	11.5	complete
PURPAN	VF CAUSSES	Latridiidae 11.1%	52	11	complete
PURPAN	PEYRIAC	Elateridae 42.9%	3	1	partial
PURPAN	CAUSSES	Curculionidae 19.4%	39	11	complete
PURPAN	PICSTLOUP	Scraptiidae 15.7%	24	4	partial
PURPAN	LUBERON201	Curculionidae 20.1%	40	10	complete
PURPAN	HTE-SAVOIE	Staphylinidae 12.4%	51	11	complete
PURPAN	RBIMAURES5	Curculionidae 17.6%	35	21	complete
PURPAN	STDAUMAS	Cerambycidae 18.2%	35	14.5	complete
PURPAN	MERCANTOUR	Cerambycidae 19.8%	45	12	complete
PURPAN	MERCAN2014	Curculionidae 18.9%	45	20	complete
PURPAN	ENTRAUNES	Curculionidae 19.2%	43	16	complete
PURPAN, EFNO	LANDES	Elateridae 18.5%	49	8	complete
SEL	Liste des espèces sensibles de Nouvelle-Aquitaine	Curculionidae 11.9%	54	5	partial
SHNAO	DREAL Auvergne	Curculionidae 21.2%	44	6	complete
SHNAO	CEN Auvergne	Curculionidae 21.2%	50	7	complete
SHNAO	Conseil Général du Cher	Curculionidae 23.2%	39	8	complete
SHNAO	RNF	Curculionidae 20.9%	41	9	complete
SHNAO	Conseil général de l'Ardèche	Cerambycidae 21.2%	42	3	partial
SHNAO	Réserve Naturelle des Gorges de l'Ardèche	Cerambycidae 48.3%	31	3	partial
SHNAO	Imerys Céramics France	Cerambycidae 15.5%	35	4	partial
SHNAO	Conseil général du Puy-de-Dôme	Cerambycidae 14.4%	51	10	complete
SHNAO	PNR Volcans d'Auvergne	Curculionidae 20.1%	33	5	partial
SHNAO	Clermont-Co	Elateridae 14.6%	19	20.5	complete
SHNAO	LPO	Curculionidae 26.4%	46	5	partial
SHNAO	R.N.F.	Elateridae 14.3%	33	3	partial
SHNAO	PNR des Volcans	Cerambycidae 22.5%	35	129	complete
SHNAO	mairie de Volvic	Curculionidae 27.2%	42	4	partial
SHNAO	Mairie de Beaumont	Ptinidae 12.2%	36	23.5	complete
SHNAO	Mairie de Romagnat	Curculionidae 15.9%	41	5	partial
SHNAO	DREAL AURA	Cerambycidae 21.2%	28	45	complete
SHNAO	SMAT	Cerambycidae 27.6%	33	2	partial
SHNAO	déparment du Puy de Dôme	Curculionidae 16.5%	44	28	complete
SHNAO	PNR Livradois-Forez	Curculionidae 18.1%	43	6	complete
SHNAO	Syndicat Mixte Monts de la Madelaine	Curculionidae 24.7%	36	17	complete
SHNAO	Syndicat des Monts de la Madelaine	Curculionidae 21.8%	31	8	complete
SHNAO	Conseil départemental d'Ardèche	Curculionidae 25.6%	41	5	partial
SHNAO	P.N.R. des Monts d'Ardèche	Elateridae 15.6%	42	12	complete
SHNAO	Conseil général d'Ardèche	Scarabaeidae 22.8%	33	19	complete
SHNAO	comcom	Curculionidae 17.6%	43	35	complete
SHNAO	BNP Paribas	Curculionidae 16%	27	75	complete
URZF	Sore	Curculionidae 39.5%	33	5	partial
URZF	Euplatypus- DSF	Cerambycidae 40.4%	10	3	partial
URZF	Portrap	Cerambycidae 57.8%	25	3	partial
URZF	Chinensis	Cerambycidae 100%	1	2	partial
URZF	Homed	Cerambycidae 50.4%	28	5	partial
URZF	sans projet	Cerambycidae 100%	1	2	partial
URZF	portrap	Cerambycidae 52.1%	11	5	partial
URZF	Bubble Canada	Cerambycidae 100%	1	4	partial
URZF	Université Padova	Cerambycidae 28.4%	30	25	complete
URZF	Monochamus	Cerambycidae 71.3%	5	5	partial
URZF	DSF	Cerambycidae 56.7%	5	3.5	partial
URZF	Anoplophora	Cerambycidae 49.8%	13	2.5	partial
URZF	Rosalia	Cerambycidae 100%	1	3.5	partial
URZF	Samfix	Cerambycidae 36.3%	25	3	partial
URZF	Aliem '	Cerambycidae 38.7%	11	5	partial
URZF	Réserves Briançonnais	Cerambycidae 55.4%	13	4	partial
